# Dr. Ryan, on Autumnal Fever

**Published:** 1803-03-01

**Authors:** Michael Ryan

**Affiliations:** Kilkenny


### *To the Editors of the Medical and Physical Journal.*

GENTLEMEN,

THOUGH it is not in my power to comply with the wish expressed by you in No. 46 of the Medical and Physical Journal, as no epidemic at present prevails in this part of the united kingdom, yet if the following observations relative to a fever that prevailed here in the autumn of 1801 and 1802, merit your attention, you may make what use you please of them.

In every autumnal season a bilious fever in one shape or other is generally found to be a very prevalent complaint in this city and the neighbourhood thereof. The fever of the last autumn not being characterized by any unusual occurrence, will not of course engage much of my attention; but the epidemic of the autumn of 1801 being accompanied with several striking peculiarities, will demand a more ample and particular consideration.

Though the last autumn was unusually healthy when compared with that immediately preceding it, several instances of an irregular intermittent fever were to be met with both in the town and in the country; and though this disorder apparently wore the garb and dress of a quotidian, it was unquestionably a tertian intermittent in disguise. After the rigor at the commencement, which was of short continuance, and never amounted to a chattering of the teeth or trembling of the limbs, the paroxysm terminated in the space of about sixteen hours, without sweating or any symptom of complete crisis, the apyrexia in general lasting the remainder of the twenty-four hours, during which time the pulse was observed to be of the natural standard and sometimes below it. The second came on, as did every paroxysm after, without horripilatio or any sense of cold whatever, and finished its course exactly in the same manner as the first. The paroxysms of the odd days were so perfectly similar to each other in every respect, and even so much more severe than those which occurred on the even days, that no doubt could be entertained of its being the double tertian described by different writers, though it put on the appearance of a quotidian ague.

After the stomach and bowels were sufficiently cleared by an emetic and laxative, recourse was immediately had to large doses of Peruvian bark, and very constantly with marked success; the general practice was to get the patient if possible to take an ounce of the powder during each intermission; and when this quantity was retained by the stomach, the fever most commonly yielded a complete victory to three or four ounces of it. In one instance the bark, instead of preventing a recurrence of the febrile attacks, operated in another way almost equally salutary; it evidently brought on a regular intermittent paroxysm, which went through the cold, the hot, and the sweating stage, and after having assumed the type of a genuine simple tertian, it was readily subdued by persevering in the use of the bark. In another instance of this irregular fever, an ounce of bark was taken every day for five days successively with no visible good effect whatever; after this the stomach rejecting it in every form, the disorder was allowed to take its course without any further interference of Art, to see if Nature would make any effort to throw it off; but after having waited for five or six days, and no hope of success appearing from this quarter, I directed an emetic to be taken as soon as the first symptom of the next return of fever should declare itself. The success exceeded expectation, a stop was put to the progress of the paroxysm by the operation of the emetic, and the patient was totally free from fever for the rest of the day; on lying down the following night some degree of chill or rigor was experienced from the cold of the sheets, and a return of fever which lasted fourteen hours was the consequence. Instructed by this event, I took particular care to have the sheets well warmed the night succeeding this; a draught or two of milk whey was in readiness to be taken as hot as it could be borne when the patient got into bed, and heated bricks were applied to the soles of the feet; the experiment succeeded most completely in keeping the paroxysm at bay, and by repeating this practice for three or four nights more, a rapid recovery took place.

About the middle of August 1801, a fever began to spread itself very generally among all ranks and descriptions of persons in this town and its vicinity, and was emphatically called the fifth day fever by the lower order of the people. This fever, at its onset, was ushered in with the symptoms usually attending other febrile disorders, and could not from any uncommon degree of severity in the cold stage be supposed to belong to the class of intermittents; it most commonly proceeded in its course in every respect like a continued fever, without any untoward or unpromising symptom (though sometimes the contrary took place) till the fifth day arrived, when it terminated by a profuse perspiration spiration and a lateritious sediment in the urine. Very often a bilious diarrhœa came on at the same time, and seemed to assist in completing the crisis, as appeared from the pulse suddenly coming down to the natural standard, in a short time after the complaint in the bowels had taken place. The patients continued in a state of apparent convalescence for four or five days, when in most cases, if unassisted by medicine, they were doomed to undergo a second attack; nor was the business then to have an end, as seldom an instance occurred when a relapse as it was called had once taken place, that a third, and sometimes a fourth return of the fever could not with a high degree of probability be counted upon. The duration of the relapse was fluctuating and uncertain, sometimes exactly measuring the same number of days as the first attack, though frequently it was observed to finish its course in the space of three days, and sometimes within the term of forty-eight hours. The interval between the paroxysm, as may be readily supposed, was not fixed or determinate, irregularities in diet and regimen, and many other incidental circumstances having often contributed to shorten the natural period; but when those adventitious causes were not allowed to operate and interrupt the regular progress of the disorder, the intermission was generally found to last from four to five days.

The pulse was in general far different to what we meet with in the typhus or low fever of this country, it was slower, fuller, and softer, and did not indicate that depression of strength and want of energy, which act so conspicuous a part in the typhus; on some occasions it is true the pulse was observed to be very frequent, feeble, and tremulous, convulsive twitchings took place in different parts of the body, and the patient was disturbed and agitated by delirium and incoherency of ideas; these however were merely the precursors of a crisis which made their appearance a few hours before this event, and even dissipated on its taking place. Somewhat about this period the crisis was now and then observed to be ushered in by a regular aguish paroxysm. After the fever had wore itself out, or was subdued by medicine, the surface of the body was often observed to be tinged with as deep a yellow colour as that of a person in a confirmed jaundice, but this appearance did not retard the patient's recovery, and gradually went off without any assistance from medicine.

When this epidemic first began to prevail, it was treated as a regular continued fever, and its terminating success-

 fully fully about the fifth day was attributed to the mode of cure that was adopted, in which James's powder and other antimonials had a considerable share; further experience soon demonstrated that this opinion was formed with rashness and precipitancy, as the issue was equally auspicious with the simple draught of Riverius as the powerful febrifuge of Dr. James.

Repeated observation having soon led to a conviction that this fever was in some respect an intermittent, very little doubt was entertained of the efficacy of the bark in effecting a cure, particularly as the intermissions were so long as to afford an opportunity of giving any quantity of it that might be thought necessary. Here also the folly of placing too much reliance on the virtue and power of medicine was apparent. It was the custom to administer an ounce of the bark in the course of twenty-four hours, and repeat it in the same quantity every day for four or five days, or as long as the intermission lasted; sometimes this practice was found to succeed effectually and prevent any further recurrence of the fever; but at other times it seemed to exert no power whatever over the disorder; and if from negligence, or any other cause, the use of the bark was not resorted to until after the conclusion of the second attack, it was then found to be of inferior force and efficacy to what it was when recourse was had to it immediately after the first paroxysm had completed its period. In this case the force of habit being superadded to the disorder, increased the power of resistance, and rendered it superior in many instances to the agency of this remedy.

Under what denomination this fever should go, and if it be placed among the intermittents, to which species it should be attached, it is not an easy matter to determine; it was certainly a continued fever for the four or five first days, and after this broke down to a type more resembling a quartan and quintan than any other. But what appears of infinitely more importance than its classification is, that of the immense numbers which during the space of almost two months were seized with this fever, scarcely an instance occurred of its proving fatal; whether the patients were treated with emetics, antimonials, &c. in the onset, and in the intermissions were liberally supplied with the bark, or that the whole business was entrusted to the vis medicatrix naturæ, the success was equally conspicuous and brilliant; this was clearly exemplified by the recovery of at least two hundred persons without medicine, without nutriment of a proper quality, without any of those comforts forts or conveniencies of life that are found to contribute so essentially to the re-establishment of the health of convalescents from fever. So fully satisfied of the inutility of medicine of every kind were the lower order of people, among which it mostly prevailed, that either owing to their daily experience of the harmless nature of the fever, to their want of faith in the efficacy of medical aid in this as well as in almost every other disease, or to both these causes united, the fact is, that no entreaty could prevail upon them to make a trial of emetics, antimonials, Peruvian bark, or any other remedy within the whole range of the Materia Medica.

From such a signal display of the salutary efforts of the constitution in the extinction of this fever, it would be wrong to argue the impotency of medicine therein, and injudicious in the extreme to reject with fastidiousness its assistance on any similar occasion in future; on the contrary, in all such cases, the cure should begin with clearing the stomach and bowels, exhibiting immediately after neutral salts and preparations of antimony, until a complete intermission be obtained, when the Peruvian bark should be given in full and efficient doses, and repeated at proper intervals, so as not to oppress and overload the stomach with two large a quantity. Though this practice may often fail in answering the sanguine expectations entertained by some persons of the infallibility of the bark in the suppression of this or a similar fever, nevertheless opportunities will frequently occur to them of beholding its prompt and decided triumph over this disease; and should it continue its course unabated, the consolation will await them of not being the authors of a more serious evil than the temporary annoyance and tumult of the stomach and bowels from the irritation of the bark.

Whether intermitting and remitting fevers be infectious or otherwise, is a question that has been much agitated among physicians, and not a little contrariety of opinion still prevails on the subject. A late eminent writer and teacher, whose opinion ought to have much weight, considered all kinds of fevers infectious, though he looked upon semitertians and other varieties of intermittents to be so in a very inferior degree. Without any partiality for either supposition, I shall relate with fidelity, what immediately fell within my own observation, and leave my readers to determine the point with themselves. I have had many opportunities of seeing single persons in different families among those in affluent circumstances, confined with the

 fever fever in question, and in no one instance could I discover that it was propagated by contagion; and from the minute investigation that took place, such an event could not have happened without my being made acquainted with it. On the other hand, whenever this fever made its appearance in a house belonging to the lower class of people, it uniformly, with very few exceptions indeed, spread itself through the whole of the inhabitants of the dwelling; to the authenticity of this fact, I can bear witness from repeated and attentive observation, and I think it can be satisfactorily explained.

The habitations of the poor and working people of this town are divided by a kind of partition into two apartments, that part which they call a kitchen, and a room immediately adjoining it, where they are all doomed to sleep, or are huddled together with scarcely a blanket to cover them. A second bed is never heard of, let the number of the family be what it may. In ninety-nine of one hundred of these hovels, neither chimney nor window is to be met with, except an aperture of about six or seven inches square, which they contrive to have glazed for the purpose of admitting light. This solicitude on their part to prevent the admission of air is for the purpose of protecting them against the inclemency of the weather during the winter, as fuel is ordinarily sold at a very advanced price at this season of the year; moreover, a hog is always an inmate of the family, and sleeps under the same roof with it every night; a heap of dung for the cultivation of potatoes, is placed on one side at the entrance of the door, from which is constantly oozing a quantity of water, which stagnates and putrifies from lodging in a bed usually dug for the reception of the manure. If any one of the family happen to be taken ill of a fever, (no house of recovery having been as yet erected for the reception of such patients) no alternative remains for the rest but to allow the sick person to remain where he is, and expose themselves to the danger of imbibing the infection that is constantly exhaling from him, until each in his turn is fated to undergo the same species of affliction and suffering as the first whose lot it was to be attacked. Under all these circumstances, it cannot be a matter of surprise or astonishment, when once a fever has got footing in one of these abodes of poverty and wretchedness, that it should prove infectious, and spread terror and dismay through the rest of the miserable inhabitants thereof. From the number of causes concurring to accumulate and concentrate concentrate the poison of infection, a person from reasoning upon the subject would be naturally led to believe, that in length of time the fever might be apt to change its nature, and assume the characteristic marks of what is called a putrid fever; but in no case whatever was such a transition found to take place; the original febrile complaint preserved its character throughout; and from the beginning to its final dissipation, the distinctive marks of an intermittent, at one period or other, were prominent and conspicuous. I am, &c.

*Kilkenny, Dec.* 11, 1802. MICHAEL RYAN, M. D.

